# Early life stress and disruption of the ageing trajectory in female rats: Insights into the origins and mechanisms underlying the risk of hypertension

**DOI:** 10.1113/EP093539

**Published:** 2026-01-31

**Authors:** Danuzia Ambrozio‐Marques, Loralie Mei Guay, Alicia A. Koogler, Tim D. Ostrowski, Aline M. Arlindo de Souza, Kathryn Sandberg, Richard Kinkead

**Affiliations:** ^1^ Research Center of the Québec Heart & Lung Institute Université Laval Québec Canada; ^2^ Department of Pediatrics, Faculty of Medicine Université Laval Québec Canada; ^3^ Department of Physiology A.T. Still University Kirksville Missouri USA; ^4^ Department of Medicine Georgetown University Washington District of Columbia USA

**Keywords:** ageing, angiotensin converting enzyme 2 (ACE2), oestrogen, hypertension, maternal separation, paraventricular nucleus of the hypothalamus

## Abstract

Menopause increases the risk of hypertension in women, yet the factors contributing to this important change remain unclear. Because early life stress has persistent and sex‐specific consequences on health, we hypothesized that ageing reveals the latent effects of neonatal maternal separation (NMS) on cardiovascular homeostasis in female rats. Following birth, rats were either subjected to NMS (3 h/day from postnatal days 3 to 12) or raised under standard conditions (CTRL). Cardiovascular and neuroendocrine functions were evaluated at three distinct ages: young adult (12 weeks), middle‐age (35 weeks) and old (64 weeks). Measurements included hormonal profile (multiplex assay), mean arterial blood pressure (MAP; tail cuff method), activity of the plasma angiotensin‐converting enzymes (ACE and ACE2), and activation of the paraventricular nucleus of the hypothalamus (PVN; FosB immunolabelling). Age‐related decline in 17β‐oestradiol (E_2_) was greater in NMS rats than CTRL. Age‐related rise in MAP was observed only in NMS; MAP was inversely correlated with E_2_ levels in NMS rats but not CTRL. In old females, ACE2 activity was 35% less in NMS than CTRL. ACE2 activity was inversely correlated with MAP in old but not young females, regardless of treatment. In the PVN, the number of FosB expressing cells decreased with age; this effect was greater in NMS females. Experiencing stress during early life is an important determinant of the ageing trajectory of females and reproductive senescence marks a turning point in regulation of cardiovascular function. Disruption of estrogen signaling and/or the renin–angiotensin system are plausible mechanisms by which NMS stress compromises cardiovascular health.

## Abstract (highlights)

- **What is the central question of this study?**
  In women, menopause increases the risk of numerous diseases, including hypertension, but not all women develop this cardiovascular problem. Why? Does experiencing stress during early life accelerates the ageing trajectory in female rats?
- **What is the main finding and its importance?**
  By comparison with unstressed rats, 1‐year‐old females previously exposed to neonatal maternal separation develop a higher blood pressure. The inverse relationship between 17β‐oestradiol levels and blood pressure observed in stressed females but not controls indicates a greater sensitivity to the age‐related drop in ovarian hormones. Prior life stresses may thus be an important risk factor in the development of hypertension in ageing women.

## 1 INTRODUCTION

Reports from the United Nations indicate that the number of individuals aged 60 years and older will reach 2.1 billion by 2050, with women representing 54% of this population (United Nations, 2019a, 2019b). Old age represents a risk factor in the development of major health issues including hypertension, metabolic disorder and sleep apnoea (Nelson, 2008; Wenger et al., 2018; Wolk et al., 2003). Hypertension is a major cause of mortality that, until recently, was often considered ‘a man's disease’ owing to its higher prevalence in young males. Yet the prevalence of hypertension in women rises rapidly following menopause and exceeds that of men at about age 60 (Drury et al., 2024; Wenger et al., 2018). Menopause is a natural process associated with complex physiological changes in women (Nelson, 2008; Wolk et al., 2003). As not all women develop these health issues, understanding the origins of this age‐related rise in hypertension specific to a subpopulation of menopausal women is necessary to address and prevent this major (and growing) disease.

In line with Barker's hypothesis of the perinatal origins of health and disease, there is robust evidence from cohort studies indicating that adverse childhood experiences increase the risk of cardiovascular disease in adulthood (Alastalo et al., 2013; Loria et al., 2013b; Murphy et al., 2017). Preclinical studies also support this concept as animal models of early life stress such as intrauterine growth restriction and neonatal maternal separation (NMS) favour the emergence of elevated blood pressure in rodents (Murphy et al., 2017). NMS is of great interest in preclinical research; unlike growth restriction or hypoxia, it poses no immediate threat to homeostasis, which facilitates disentangling the effects of activating the neuroendocrine stress response during a critical developmental period from the direct impacts of stress on the organism.

In mammals (including humans), the tactile, olfactory and auditory stimuli provided by the mother are among the most potent factors influencing brain development in the newborn (Gunnar, 2003; Nicolson, 2004; Parker et al., 2006). Consequently, adverse conditions such as an unstable or inadequate parental environment and specialized medical care following birth, which interfere with mother–infant interactions, compromise neurological outcomes (Battaglia et al., 2009; Gunnar, 2003; Shonkoff et al., 2009). The mechanisms by which disrupted maternal care alters developmental trajectories remain a fundamental issue, and it is now well established that mother–infant interactions contribute to programming the stress‐related neuroendocrine pathways in the offspring. Several laboratories (including ours) have shown that abnormal or insufficient maternal interactions disrupt the balance between excitatory and inhibitory mechanisms regulating the paraventricular nucleus of the hypothalamus (PVN), a structure that orchestrates the release of stress peptides and hormones (Tenorio‐Lopes & Kinkead, 2021; Ulrich‐Lai & Herman, 2009). Inadequate maternal care therefore increases basal activity of the hypothalamic–pituitary–adrenal axis and potentiates stress responsiveness throughout life (Caldji et al., 2000). Much like chronic stress, this condition elevates the risk for a broad range of health issues, including diabetes and cardiovascular disease (Lombard, 2010; McEwen & Gianaros, 2010; Pietrobon et al., 2020).

Interestingly, some studies report that early life stress has a greater effect in males than females (Genest et al., 2004) with sex‐specific mechanisms (males only) including NMS‐related increase in carotid body function (Soliz et al., 2016), activation of the stress PVN with enhanced basal corticosterone levels (Elliot‐Portal et al., 2021; Genest et al., 2004), and renal dysfunction (Loria et al., 2013a). Once they reach ~1 year old, however, NMS females show physiological anomalies in line with those reported in sleep apnoea patients, including a higher apnoea index during sleep, excessive responsiveness to respiratory challenges, and higher body mass index with augmented proportion of body fat (Ambrozio‐Marques et al., 2026; Fournier et al., 2015). These results are consistent with other preclinical studies indicating that the progressive loss of the neuroprotective and anti‐inflammatory effects of ovarian hormones contribute to age‐related onset of cardiovascular issues (Laouafa et al., 2019; Lima et al., 2012; Wolk et al., 2003). As sleep apnoea and obesity are major comorbidities of hypertension, we first tested the hypothesis that ageing reveals the latent effect of NMS on cardiovascular homeostasis. The blood and brain samples obtained at the end of the experiments allowed us to evaluate the impact of NMS on key neuroendocrine mechanisms regulating cardiovascular function. We then focused on how NMS and ageing affect the activity of angiotensin converting enzyme (ACE) and angiotensin converting enzyme II (ACE2), as this key element of the renin–angiotensin system contributes to sex‐based differences in hypertension (Drury et al., 2024). We then used FosB immunolabelling to compare basal activation of the paraventricular nucleus of the hypothalamus (PVN), an important structure in the regulation of blood pressure and neuroendocrine response to stress; previous work shows that in adults, NMS augments basal PVN activity in a persistent and sex‐specific fashion (Tenorio‐Lopes & Kinkead, 2021).

## 2 METHODS

### 2.1 Ethical approval and animal housing

All protocols were conducted in accordance with guidelines outlined by the Canadian Council on Animal Care and approved by the Laval University Animal Care Committee (protocol no. 2020–441). Descriptions of the key information regarding animal use and experimental protocols are in line with the ARRIVE guidelines 2.0 and the guidelines of the Canadian Council on Animal Care. All animals were born and raised in the animal facilities of the Québec Heart and Lung Institute Research Centre (Quebec City, Canada) under standard laboratory conditions with a 12‐h light–dark cycle (lights on at 07.00 h), controlled temperature (22 ± 1°C) and humidity (55%). Food and water were provided ad libitum throughout the duration of the study. Adult male and nulliparous female Sprague–Dawley rats were obtained from Charles River Laboratories (Montréal, Canada) for reproduction. After confirmation of mating, pregnant females were individually housed and monitored daily until gestational day 21 when birth typically occurred, resulting in litters of 12–15 pups. When necessary, litters were standardized within 48 h of birth to 12 pups per dam, with balanced male‐to‐female ratios, to reduce variability due to litter size and sex composition. At the end of the experiments, rats were deeply anaesthetized by a terminal intraperitoneal injection of a mixture of ketamine (80 mg kg^−1^; Pfizer, Kirkland, QC, Canada) and xylazine (10 mg kg^−1^, Bimeda, Cambridge, ON, Canada).

### 2.2 NMS protocol

As previously described in our group's protocols (Ambrozio‐Marques et al., 2026; Genest et al., 2007; Tenorio‐Lopes et al., 2017), pups were randomly assigned to either Control (CTRL) or NMS conditions. In the NMS group, the entire litter was separated from the mother and placed into individual compartments of a humidity‐controlled incubator (33°C, 45% relative humidity) for 3 h/day (09.00–12.00 h) from postnatal day (P)3 to P12. After P12, both NMS and CTRL litters remained undisturbed with the dam until pups were weaned at P21. Following weaning, female pups were housed in pairs with littermates from the same experimental group. Notably, male offspring were excluded from the present study and used for other research projects within our laboratory. To avoid confounding litter‐specific effects, animals from at least three different litters were included in each experimental group. All experimental procedures were conducted between 09.00 h and 13.00 h; blood and brain samples were obtained during the same period to minimize the influence of circadian fluctuations on physiological parameters.

### 2.3 Age groups

**FIGURE 1:**
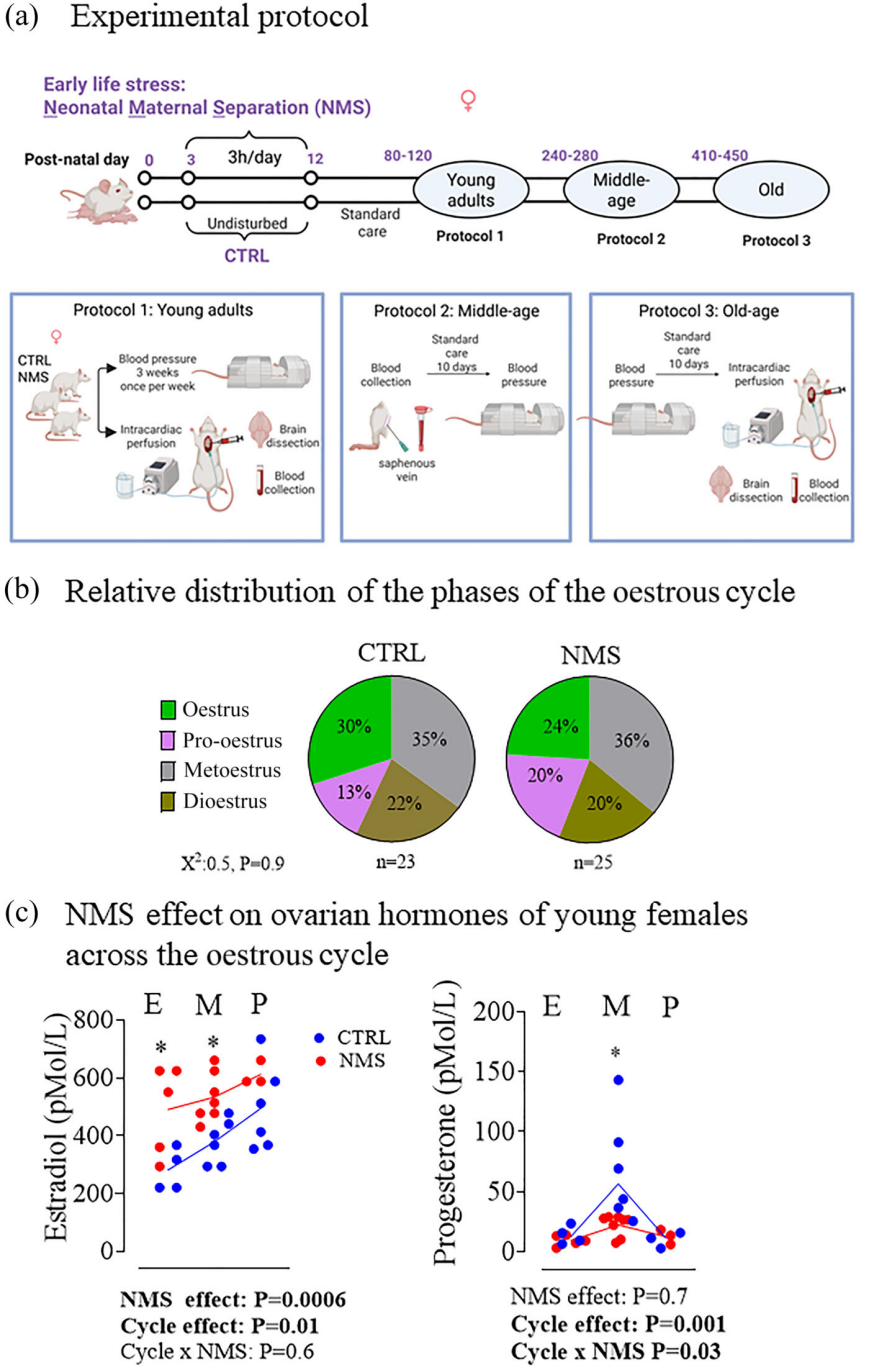
(a) Top: timeline of the study design highlighting the age groups and experimental treatments. Bottom: schematic representation of the experimental procedures performed in each age group. Created with BioRender. (b) Pie charts comparing the relative distribution of the phases of the oestrous cycle of young females either subjected to neonatal maternal separation protocol (NMS; 3 h/day from postnatal days 3 to 12) or maintained under standard animal care over the same period (control; CTRL). Distribution was compared with a chi‐square test. (c) Comparison of circulating levels of 17β‐oestradiol (left) and progesterone (right) in NMS (red) and CTRL (blue) in young females during the oestrus (E), metoestrus (M), and pro‐oestrus (P) phases of the oestrous cycle. Note that dioestrus was not tested as this stage shares similar hormonal profiles with metoestrus (low E_2_ and increasing progesterone). ANOVA results are reported under each figure. *Mean value significantly different from the corresponding NMS value at *P* < 0.05, and significant *P*‐values are highlighted in bold.

To investigate how early‐life stress affects the ageing trajectory of females, experiments were performed at three key stages of the female rat's reproductive lifespan (Figure 1). These stages were chosen based on previous work characterizing the physiological and hormonal changes that occur as rats transition from regular reproductive function to reproductive senescence (Diaz Brinton, 2012; Fournier et al., 2015; Nass et al., 1984). (1) The young adult group (Young) included female rats between P80 and 120 (~12–17 weeks old) when rats show regular oestrous cycles and well‐regulated secretion of ovarian hormones; this is the only group in which the oestrous cycle was determined. (2) The middle‐aged group (Middle‐age) included rats between P240 and 280 (~35–40 weeks old). This period corresponds to the transition between regular reproductive function and reproductive decline, similar to perimenopause in women (Diaz Brinton, 2012; Nass et al., 1984). (3) The old group (Old) included animals aged P410–450 (~59–64 weeks old). At this age, most females are no longer cycling and are considered reproductively senescent. Reproductive senescence in rats is often used as a model for menopause‐like conditions in humans (Diaz Brinton, 2012; Fournier et al., 2015; Nass et al., 1984).

While ovariectomy (OVX) is the gold standard in preclinical research for evaluating the effects of gonadal hormones in females (Koebele & Bimonte‐Nelson, 2016), OVX in young rats does not mimic natural ovarian senescence because of the age difference and the continued presence of ovaries. Moreover, studies show that the impact of OVX on respiratory reflexes differs from that of natural ageing, especially in NMS females (Grittner et al., 2025).

### 2.4 Experimental design

As illustrated in Figure 1, animals were randomly distributed into experimental groups based on two main factors: (1) exposure to early life stress (CTRL vs. NMS), and (2) age group (Young, Middle‐age or Old). The experimental assessments in each age group were adapted according to the invasiveness of tissue sampling. To enable longitudinal analysis, the experimental cohort initially included 30 female rats. Of these, 15 animals were assessed during early adulthood and subsequently sacrificed for tissue and blood collection. The remaining 15 animals were maintained and monitored longitudinally. For middle‐aged animals, brains were not harvested, but a small blood volume was collected from the saphenous vein (non‐terminal), followed 10 days later by a blood pressure measurement. Females were then maintained in standard housing until they reached old age. The small volume collected in middle age females was used for hormone analysis but was not sufficient for enzymatic analyses (ACE and ACE2).

Once females reached old age, blood pressure was measured and a 10‐day recovery period was allowed before they were deeply anaesthetized for terminal blood and brain sampling. Plasma samples were used to measure ACE and ACE2 activity, circulating levels of 17β‐oestradiol (E_2_), progesterone, adrenocorticotropic hormone (ACTH) and corticosterone. Brains were processed for immunohistochemical analysis (details below).

### 2.5 Evaluation of oestrous cycle

The oestrous cycle was determined only in young females. At this age, the oestrous cycle of rats consists of four distinct phases, oestrus, metoestrus, dioestrus and pro‐oestrus, which were identified by vaginal cytology as previously described (Marques et al., 2015). The cycle phase was registered and correlated with hormonal status, blood pressure and other outcomes. The oestrous cycle was not assessed in middle‐aged females as their cycle is irregular owing to disruptions in the secretion of gonadotropins (Diaz Brinton, 2012; Nass et al., 1984). As vaginal smears can be stressful, especially to NMS females due to their increased responsiveness to adverse stimuli (Pierce et al., 2014), blood pressure measurements were performed before the assessment of the oestrous cycle.

### 2.6 Cardiovascular measurements

Arterial blood pressure and heart rate (HR) were measured in conscious, restrained animals using a non‐invasive tail‐cuff plethysmography system (CODA System, Kent Scientific, Torrington, CT, USA). This indirect method records systolic blood pressure (SBP), diastolic blood pressure (DBP), mean arterial pressure (MAP) and HR via a volume–pressure recording sensor. This method is recommended by the American Heart Association (Bogdan et al., 2019; Feng et al., 2008). Briefly, animals were placed inside restraining tubes positioned over a heated platform for 1 h to acclimatize. For each animal, 10 recordings were taken at 30‐s intervals. The average of these 10 values was used in the results. In the young group, animals were subjected to weekly blood pressure measurements for three consecutive weeks to ensure full acclimatization and to capture readings across different phases of the oestrous cycle.

### 2.7 Blood sampling and hormone analysis

Blood was collected into EDTA‐treated microtubes. Samples were kept on ice and centrifuged at 10,000 *g* for 10 min at 4°C. The resulting plasma was carefully separated, aliquoted and stored at –80°C until analysis. To reduce the risk of experimental manipulation interfering with hormone values, blood and brain samples were obtained 1 week after the last blood pressure measurement (see details below).

Plasma concentrations of ovarian and stress‐related hormones were quantified using a multiplex bead‐based chemiluminescence immunoassay system (Luminex 200, Bio‐Rad Laboratories Inc., Hercules, CA, USA), following the manufacturer's instructions. A minimum of 50 beads per analyte was acquired, and standard curves were calculated using a five‐parameter logistic curve‐fitting model (Scheinert et al., 2015). All assays were performed using commercial kits supplied by EMD Millipore (Billerica, MA, USA). The following hormones were measured: 17β‐oestradiol (E_2_) and progesterone, using the Multi‐Species Hormone Magnetic Bead Panel (cat. no. MSHMAG‐21K), and ACTH and corticosterone, using the Milliplex Rat Stress Hormone Magnetic Bead Panel (cat. no. RSHMAG‐69K‐02). Due to sample volume limitations, ovarian hormones were assessed in all age groups, whereas ACTH and corticosterone levels were measured only in young and old animals. To reduce assay costs, hormonal analyses were performed on only three stages (oestrus, pro‐oestrus and metoestrus). Dioestrus was not tested as this stage shares similar hormonal profiles with metoestrus (low E_2_ and increasing progesterone).

### 2.8 Brain immunohistochemistry

#### 2.8.1 Brain collection and sectioning

Once animals reached a deep plane of anaesthesia after the terminal ketamine–xylazine injection, intracardiac perfusion was performed using 0.9% saline followed by 4% paraformaldehyde (PFA; 1 mL/g body weight) in 0.1 M sodium tetraborate buffer (pH 7.4 at 4°C). Brains were dissected and post‐fixed in PFA for 24 h, then transferred to a 30% sucrose–PFA solution for 48 h at 4°C for cryoprotection. Samples were then frozen on dry ice and stored at −80°C until they were used. Coronal sections (40 µm thick) were cut using the dry ice method (Anders, 1942). Sections were stored at −20°C in cryoprotectant solution (0.05 M sodium phosphate buffer, 30% ethylene glycol, 20% glycerol) until further processing.

#### 2.8.2 Immunohistochemical labelling for FosB, synaptophysin and glial fibrillary acidic protein

Free‐floating immunohistochemistry was performed for the detection of FosB, synaptophysin and glial fibrillary acidic protein (GFAP), following validated protocols (Ansorg et al., 2015; Humphrey et al., 2023; Nestler, 2012). Our analyses were performed in the PVN (bregma: −1.80 to −1.88) based on anatomical coordinates defined by Paxinos and Watson's stereotaxic atlas (Paxinos & Watson, 1998). The PVN was selected owing to its contribution to cardiovascular homeostasis and its role in regulation of the stress‐related neuroendocrine pathways (Ambrozio‐Marques et al., 2023; Handa et al., 1994; Oyola et al., 2017; Tenorio‐Lopes & Kinkead, 2021). All antibodies were validated by confirming staining patterns consistent with those reported in the literature and by omitting the primary antibody, which resulted in no fluorescent signal (negative control) (Ansorg et al., 2015).

##### FosB immunolabelling

FosB is a transcription factor regulated by neuronal activity (Nestler, 2012). The expression of FosB is slower (peaks after 6 h) than other commonly used immediate early genes (e.g. cFos which peaks after 2 h; Nestler et al., 2001). Consequently, quantification of the number of FosB expressing perikarya is a well‐established marker of sustained neural activation associated with chronic rather than acute stress exposure. FosB immunolabelling in the PVN was performed according to established procedures in our laboratory (Ambrozio‐Marques et al., 2023). Briefly, FosB antigen retrieval was carried out by incubating sections in 0.1 M citric acid and 0.1 M sodium citrate (pH 6.0) at 65°C for 5 min and then at 95°C for 10 min. After cooling to room temperature, endogenous peroxidase activity was blocked using 3% H_2_O_2_ in Tris‐buffered saline (TBS) for 30 min, followed by a blocking step with 1% BSA and 0.4% Triton X‐100 in TBS. Sections were incubated overnight at 4°C with anti‐FosB primary antibody (1:500; Cell Signaling Technology, Danvers, MA, USA cat. no. 2251). On the following day, sections were incubated with biotinylated goat anti‐rabbit secondary antibody (1:400; Vector Laboratories, Burlingame, CA, USA), followed by ABC complex and 3,3′‐diaminobenzidine (DAB)–nickel reaction (SigmaFast DAB with metal enhancer, Sigma‐Aldrich, St Louis, MO, USA) for 4 min to reveal the biotinylated antibody.

After a final series of TBS washes (three 5‐min washes), the sections were mounted onto Fisherbrand Tissue Path Superfrost Plus Gold slides (Thermo Fisher Scientific, Waltham, MA, USA), dried for 48 h, and coverslipped with a permanent mounting medium, preparing them for microscopic examination. Slides with the FosB immunolabeled tissue were visualized under a microscope (Eclipse E600, Nikon, Tokyo, Japan) equipped with a camera (Infinity 3, Lumenera Corporation, Ottawa, ON, Canada) and stored as digital images. Images were digitally acquired into using Fiji (ImageJ v1.53t, NIH, Bethesda, MD, USA).

For quantification of immunoreactive cells, counting was performed within standardized regions of interest (Figure 5b) placed in the parvo‐ and magnocellular regions of the PVN. Magnocellular neurons are large (13−19 µm) neurosecretory cells that project to the posterior pituitary, including neurosecretory cells that release vasopressin, which influences blood pressure. Parvocellular neurons are small neurons (6−10 µm) with distinct phenotypes and projections including the median eminence, where they secrete numerous hormones and neurotransmitters; their influence on blood pressure is via pre‐autonomic actions (Sladek et al., 2015). Some parvocellular neurons exert a pre‐autonomic influence owing to its projections to the brainstem and spinal cord (Sladek et al., 2015). Results as expressed in cell density (cells/area). All quantifications were conducted in a randomized order by two independent experimenters who were blinded to the experimental conditions. Left and right hemispheres were both analysed and averaged, as no significant lateralization was observed in preliminary statistical testing (one‐way ANOVA).

##### Synaptophysin and GFAP staining

Within the brain, synaptophysin is a widely expressed protein in presynaptic terminals of neuroendocrine cells and neurons. GFAP was labelled to identify and visualize astrocytes, a type of glial cell, in tissue samples. Immunolabelling of those proteins offers a simple way to evaluate the long‐term impact of early life stress on PVN circuit functionality. For fluorescence labelling of synaptophysin and GFAP (Humphrey et al., 2023), sections were blocked with 10% normal donkey serum in 0.3% Triton X‐100–phosphate‐buffered saline (PBS) and incubated overnight with primary antibodies: synaptophysin 1 (guinea pig, 1:1000; cat. no. 101004, Synaptic Systems, Göttingen, Germany, RRID: AB_1210382) and anti‐GFAP (chicken, 1:4000; cat. no. GFAP, Aves Labs, Davis, CA, USA, RRID: AB_2313547). After primary antibody incubation, sections were washed and incubated for 2 h with the corresponding secondary antibodies conjugated to fluorophores, all from Jackson ImmunoResearch Laboratories (West Grove, PA, USA), and diluted at 1:200: Alexa Fluor 488 donkey anti‐guinea pig (cat. no. 706‐546‐148, RRID: AB_2340473) and Alexa Fluor 647 donkey anti‐chicken (cat. no. 703‐605‐155, RRID: AB_2340379).

Sections were mounted using ProLong Diamond Antifade Mountant (cat. no. P36962, Thermo Fisher Scientific) and stored in the dark until imaging. Images were acquired using a BX51WI fluorescence microscope (Olympus) equipped with a pE‐300 white LED light source (CoolLED Ltd., Andover, UK) and an optiMOS camera (QImaging, Surrey, BC, Canada). Due to logistic problems, immunohistochemical assays could not be performed at the same time for all groups. To adjust data for possible inter‐run variability, fluorescence intensities were normalized/scaled using control sections from two young and two old samples that were re‐stained together. Images were processed and analysed using Fiji. Results are expressed in raw fluorescence intensity values.

### 2.9 ACE and ACE2 activity

ACE and its homologue ACE2 play opposing roles in the renin–angiotensin system and blood pressure homeostasis (Figure 7a). By converting angiotensin I to angiotensin II, ACE favours vasoconstriction. Conversely, ACE2 converts angiotensin II to angiotensin 1–7 and thus promotes vasodilation. These proteins were assessed using a fluorometric assay based on the hydrolysis of specific fluorogenic substrates, as previously described (Ji et al., 2020). Briefly, assays were performed in 96‐well black microplates (Greiner Bio‐One, Frickenhausen, Germany), with a final reaction volume of 100 µL per well. The enzymatic reaction was carried out at 37°C using a buffer composed of 75 mmol/L Tris–HCl (pH 7.5), 1 mol/L NaCl, and 0.1 mmol/L ZnCl_2_. ACE and ACE2 activities were analysed in separate assay runs.

For ACE activity, 1 µL of plasma was incubated with 10 µL of 60 µmol/L *o*‐aminobenzoic acid (Abz)‐Phe‐Arg‐Lys (Dnp)‐Pro‐OH (Enzo, Farmingdale, NY, USA). The increase in fluorescence, resulting from the cleavage of the substrate and separation of Abz from 2,4‐dinitrophenyl (Dnp), was monitored over time using a FLUOstar Omega plate reader (BMG Labtech, Cary, NC, USA), with excitation at 320 nm and emission at 410 nm. Total ACE activity was calculated from the slope of the linear phase of the fluorescence curve (between 40 and 100 min), and non‐specific activity was determined in the presence of the ACE inhibitor captopril (30 µM/L; Sigma‐Aldrich). Specific ACE activity was calculated by subtracting non‐specific from total activity.

For ACE2, 10 µL substrate [Mca‐Ala‐Pro‐Lys(Dnp) (Enzo)] was added at a final concentration of 30 µM, plus 5 µL of plasma and 80 µL of the reaction buffer. For total activity, 5 µL of captopril (30 µM, Sigma) was added to the reaction buffer. For non‐specific activity, 5 µL of captopril and 5 µL of MLN‐4760 (30 µM; Millennium Pharmaceuticals, Cambridge, MA, USA) were added to the reaction buffer. The specific signal was defined by subtracting the total activity minus the non‐specific reaction, adapted from Ji et al. (2020). Measurements were performed in duplicate for each animal, and values were averaged for statistical analysis. Because samples of young and old animals were collected and processed at different time points, this difference in readings may reflect either an intrinsic age‐related change or a batch‐related artifact. To ensure data consistency, all assays were conducted under identical conditions – including substrate concentration, buffer composition and detection wavelength, thus minimizing inter‐assay variability.

### 2.10 Statistics

The effects of NMS on the relative distribution of the oestrous cycle phases were tested using the χ^2^ test. A two‐way ANOVA was performed to analyse main effects of treatments (CTRL vs. NMS) and ageing. Because our experimental design made it possible to measure cardiovascular variables of a subgroup of animals longitudinally across the different age groups, a two‐way repeated‐measures ANOVA was conducted to assess the effects of treatment and age. This design also allowed us to evaluate age‐related changes in cardiovascular variables by using the young group as a reference and express values obtained in older animals as a percentage change from young (Figure 3c). When factorial effects (or interactions) were significant, *post hoc* comparisons were made using the Holm–Šidák multiple comparisons test (*P* < 0.05). To evaluate the potential influence of the oestrous cycle on blood pressure and HR in young females, a two‐way ANOVA was performed with stress (CTRL vs. NMS) and cycle phase (oestrus, metoestrus, dioestrus and pro‐oestrus) as factors. For correlation analyses (Figure 8), Pearson's correlation coefficient (*r*) was calculated to assess the linear relationship between variables; the coefficient of determination (*r* ^2^), indicating the proportion of the variance in the dependent variable of interest that can be explained by the independent variable, is reported in the figures. The significance of each correlation was tested using a two‐tailed *P*‐value, with statistical significance set at *P* < 0.05. Results from correlation analyses were validated with an analysis of covariance (ANCOVA) with treatment and age as main factors and hormone levels as a covariate. Because samples of young and old animals used for analysis of ACE and ACE2 activity were collected and processed at different time points, the effect of NMS on activity values was tested separately in each age group with Student's unpaired *t*‐test (Figure 7). All statistical analyses were conducted using Prism (Version 8.4.2, GraphPad Software, San Diego, CA, USA). Note that to facilitate reading and improve clarity, the results of ANOVA and *post hoc* tests are reported in the figures.

## 3 RESULTS

### 3.1 NMS affects circulating hormone levels but not oestrous cycle in young females

Young females are the only group with a regular oestrous cycle and the relative distribution of the cycle phases observed at sampling did not differ between NMS and CTRL (Figure 1b). In these animals, E_2_ and progesterone levels changed across cycle phases; as expected, E_2_ peaked during pro‐oestrus whereas progesterone peaked during metoestrus (Figure 1c). 17β‐Oestradiol levels of NMS females were, on average, 25% higher than CTRL across all cycle phases (Figure 1c). For progesterone, the values obtained in NMS females differed from CTRL only during metoestrus when they were 65% lower than CTRL. Note that because metoestrus and dioestrus share very similar hormonal profiles, hormonal analyses were not performed on samples from females in dioestrus (see Methods).

### 3.2 Age and NMS‐related changes in ovarian hormones

**FIGURE 2:**
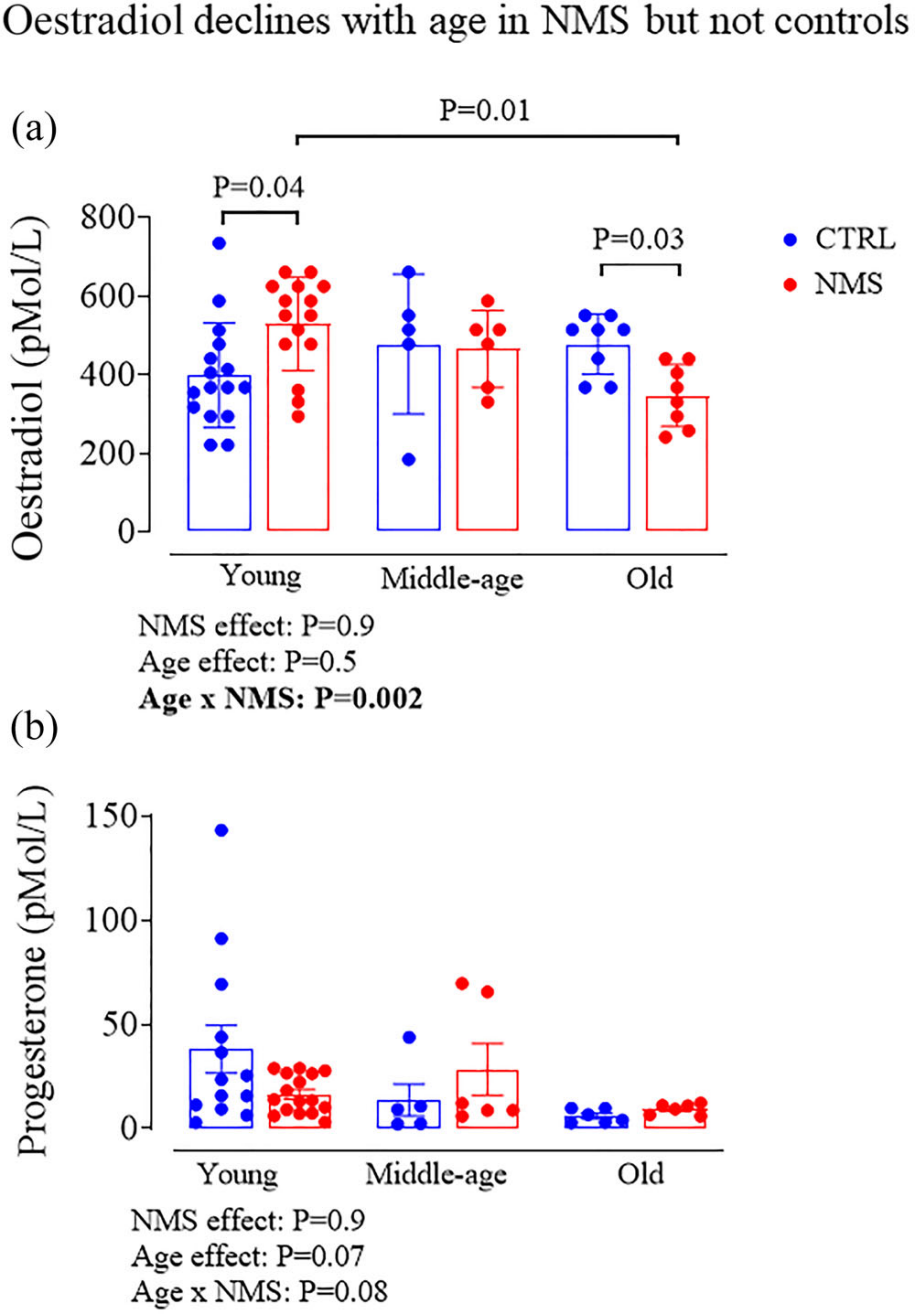
Comparison of the age‐related changes in plasma 17β‐oestradiol (a) and progesterone (b) between females either subjected to neonatal maternal separation protocol (NMS, red symbols; 3 h/day from postnatal days 3 to 12) or maintained under standard animal care over the same period (control; CTRL, blue symbols). Note that for young females, values from the different oestrous cycle phases were pooled. Bar height indicates mean value ± SD. ANOVA results are reported under each figure and significant *P*‐values are highlighted in bold.

To compare hormone levels between age groups, values obtained during the oestrus phase of young females were pooled. 17β‐Oestradiol concentrations of CTRL animals remained stable across ages but dropped progressively with age in NMS females (Figure 2a). By comparison with CTRL, the 32% higher E_2_ values measured in young NMS females contribute to this effect; by old age, E_2_ levels were 27% lower than in CTRLs. Despite suggestive trends, the age‐related decline in progesterone was not significant; the effect of NMS exposure on progesterone was not significant (Figure 2b).

### 3.3 Age‐related increase in blood pressure in NMS but not CTRL

**FIGURE 3:**
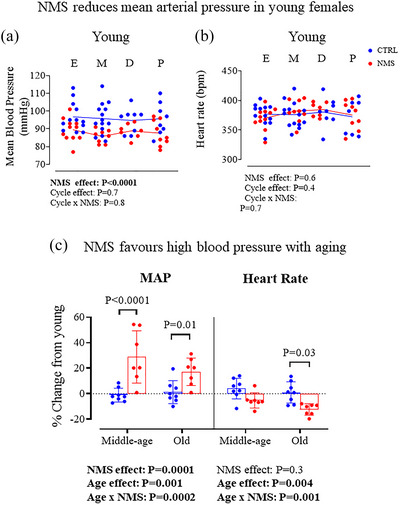
(a, b) Comparison of mean arterial pressure (MAP) (a) and heart rate (b) values across the oestrous cycle phases in young females (oestrus, metoestrus, dioestrus and pro‐oestrus). Data are reported for females that were subjected to neonatal maternal separation protocol (NMS, red symbols; 3 h/day from postnatal days 3 to 12) or maintained under standard animal care over the same period (control; CTRL, blue symbols). (c) Age‐related changes in MAP and heart rate in CTRL and NMS females. For each animal, data are expressed as a percentage change from the corresponding mean value measured when the animal was young. Bar height indicates mean value ± SD. ANOVA results are reported under each figure. *P*‐value for the *post hoc* test is indicated in the figure and significant *P*‐values are highlighted in bold.

In young females, the MAP and HR values did not differ significantly across the different phases of the oestrous cycle (Figure 3a, b). However, the MAP was 11% lower in NMS females compared to CTRLs (Figure 3a); HR was similar between groups (Figure 3b). Comparable results were obtained for the systolic and diastolic pressure (data not shown). The last values obtained in young females (week 3) are reported in Table 1 and used for comparisons with older age groups. Expressing these results relative to the values obtained in young animals showed that ageing was associated with a significant rise of MAP in NMS but not CTRL (Figure 3c, left). By comparison with young females, the MAP measured at middle age was augmented by 21% and 15% at old age. Statistical analysis of absolute (non‐normalized) values yielded similar results (Table 1). These rises in MAP were associated with HR reductions of 5% at middle age and 12% at old age (Figure 3c, right; Table 1).

**TABLE 1:** Comparison of the main cardiovascular variables measured in females either raised in standard conditions (control) or subjected to neonatal maternal separation (NMS; 3 h/day from postnatal days 3 to 12).

	Control			NMS			ANOVA results		
	Young	Middle‐age	Old	Young	Middle‐age	Old	NMS effect	Age effect	Interaction
Mean arterial pressure (mmHg)	100 ± 10	93 ± 4	95 ± 3	**90 ± 7^#^**	**114 ± 11^#,a^**	**105 ± 6^#,a^**	***P* = 0.0001 *F*_(1,26) _= 29.7**	***P* = 0.001 F_(2,26) _= 10.1**	***P* = 0.0002 F_(2,26) _= 11.9**
Heart rate (bpm)	378 ± 27	374 ± 20	363 ± 23	377 ± 19	359 ± 24	**332 ± 18^#,a,b^**	*P* = 0.3 *F*_(1,26) _= 1.1	***P* = 0.004 F_(2,26) _= 7.7**	***P* = 0.001 F_(2,26) _= 8.9**

Measurements were performed on the same animal at three different ages. Data are reported as means ± standard deviation. Significant differences between means are indicated in bold (*P* < 0.05) with # indicating a value different from corresponding control value, ‘a’ different from young and ‘b’, different from middle age.

### 3.4 Ovarian hormone levels correlate with MAP in NMS females but not CTRL

**FIGURE 4:**
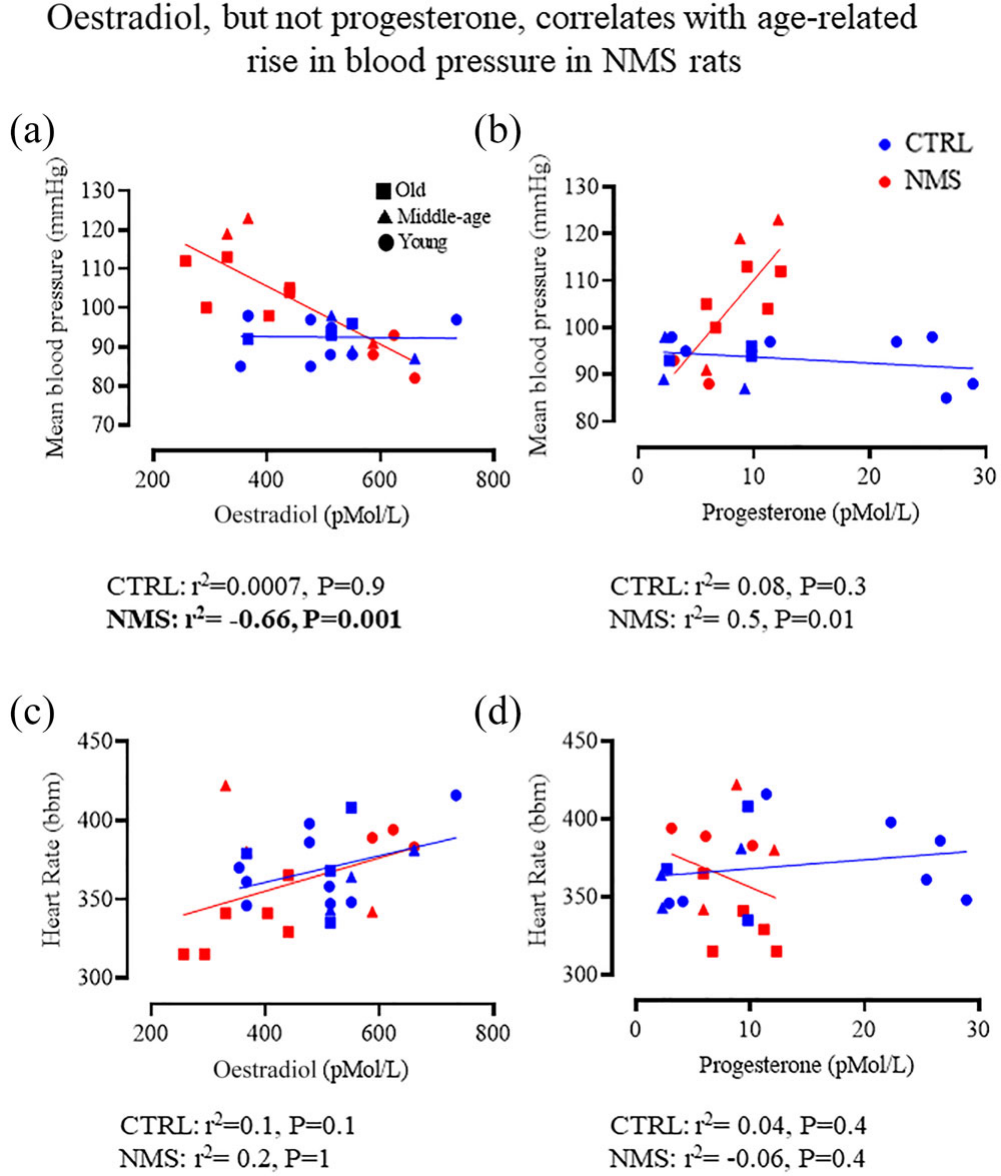
Correlation analyses of the relationships between (a) mean arterial pressure and 17β‐oestradiol, (b) mean arterial pressure and progesterone, (c) heart rate and 17β‐oestradiol, and (d) heart rate and progesterone. Data are reported for females subjected to neonatal maternal separation protocol (NMS, red symbols; 3 h/day from postnatal days 3 to 12) or maintained under standard animal care over the same period (control; CTRL, blue symbols). The origin of the age group of each value is indicated by a distinct symbol: ● young, ▲ middle‐age, and ■ old. Coefficients of determination (*r* ^2^) and *P*‐values are reported separately for CTRL and NMS. Significant *P*‐values are highlighted in bold.

In animals and humans, drops in ovarian steroids are generally associated with a rise in blood pressure, but for reasons that are still unclear, these effects are highly heterogeneous (Drury et al., 2024). Correlation analysis showed that in NMS females, lower E_2_ concentrations were strongly associated with higher blood pressure but no relationship was observed in CTRL (Figure 4a). Of note, the low E_2_ values originate mainly from middle‐age and older females. ANCOVA supports this observation as indicated by a significant interaction between NMS exposure and E_2_ levels (*F*_1_,_22_ = 8.3, *P* = 0.009) as well as Age vs. E_2_ (*F*_1_,_19_ = 8.1, *P* = 0.003). In contrast, progesterone levels were positively associated with MAP in NMS females but not CTRL (Figure 4b). ANCOVA revealed a significant interaction between stress and progesterone levels in predicting MAP (*F*_1_,_17_ = 7.7, *P* = 0.01), as well as the interaction among Age vs. Stress vs. Progesterone in predicting MAP (*F*_1,11_ = 3.7, *P* = 0.05). Correlations between HR and either E_2_ or progesterone levels were not significant (Figure 4c and d, respectively).

### 3.5 Impact of NMS on stress‐related hormones and neuronal markers

#### 3.5.1 Ageing augments ACTH, but not corticosterone levels

**FIGURE 5:**
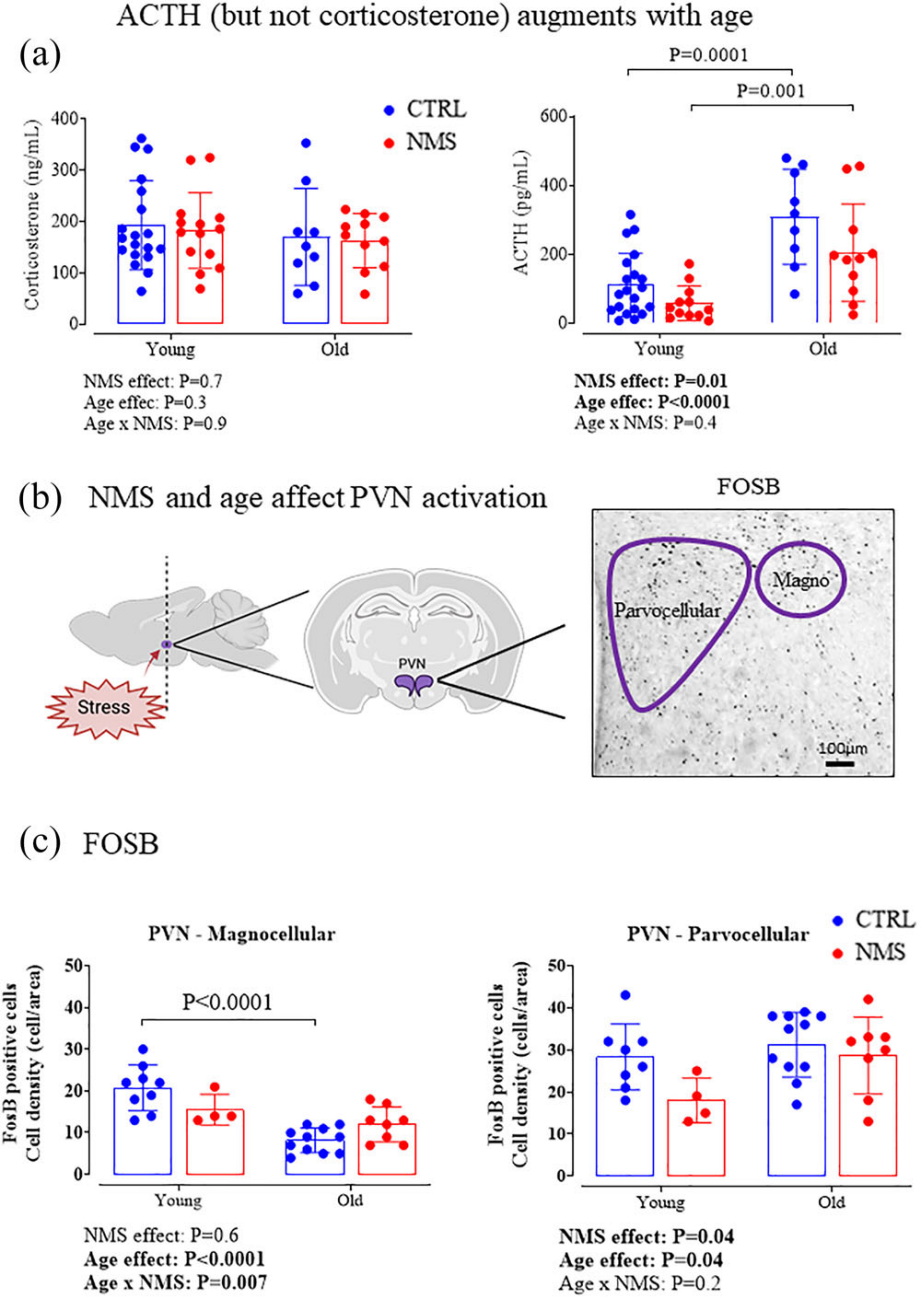
Effects of age and neonatal maternal separation (NMS) on the hypothalamic–pituitary–adrenal axis. (a) Comparison of plasma levels of corticosterone and adrenocorticotropic hormone (ACTH) measured in young or old female rats that were subjected to the NMS protocol (red symbols; 3 h/day from postnatal days 3 to 12) or maintained under standard animal care over the same period (control; CTRL, blue symbols). (b) Schematic representations of the anatomical location of the paraventricular nucleus of the hypothalamus (PVN) which orchestrates the neuroendocrine response to stress. Left: sagittal view of the rat brain indicating the location of the PVN; the dashed line indicates the level at which tissue sections were obtained for immunohistochemical analysis. Middle: schematic representation of a frontal section indicating the location of the PVN. Right: enlarged photomicrograph illustrating FosB immunolabelling in the PVN; the parvo‐ and magnocellular regions where FosB expressing perikayas were counted are drawn on the image. (c) Quantification of FosB immunolabelling in the magno (left) and parvo (right) subdivisions of the PVN in CTRL and NMS females from the young and old group. Data are expressed as cell density. Bar height indicates mean value ± SD. ANOVA results are reported under each figure and significant *P*‐values are highlighted in bold.

At young age, oestrous cycle did not affect stress hormone levels in either group (data not shown; cycle effect: *P* = 0.1 and 0.8; treatment effect: *P* = 0.5 and 0.4; cycle × treatment: *P* = 0.8 and 0.9 for corticosterone and ACTH, respectively). Thus, data from young females obtained during different phases were pooled for subsequent comparisons with old females. Corticosterone concentrations were not affected by NMS or age (Figure 5a, left). In contrast, ACTH levels were lower in NMS than CTRL; the age‐related rise in levels was similar in both groups (2.8‐ and 3.5‐fold for CTRL and NMS, respectively; Figure 5a, right).

#### 3.5.2 NMS and ageing modulate FosB expression within the PVN

Quantification of FosB‐expressing cells in the two PVN subregions (Figure 5b) revealed distinct age‐ and stress‐related patterns (Figure 5c). In the magnocellular PVN, ageing markedly reduced FosB expression by 60% in CTRL females, whereas the decline was modest in NMS animals (23% decrease). In the parvocellular PVN, both age and NMS affected FosB expression; however, their effects were not interrelated. The density of FosB expressing cells of young NMS females was 36% less than CTRL, but expression increased with age, whereas it remained stable in CTRL.

#### 3.5.3 Synaptophysin and GFAP expression in the PVN over age

**FIGURE 6:**
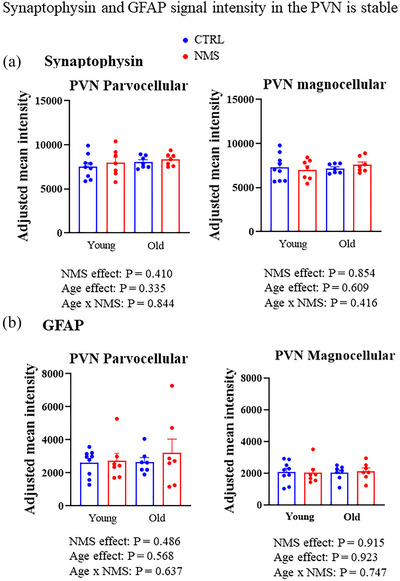
Effects of age and neonatal maternal separation (NMS) on the intensity of (a) synaptophysin and (b) glial fibrillary acidic protein (GFAP) immunolabelling in the parvocellular (left) and magnocellular (right) subdivisions of the paraventricular nucleus of the hypothalamus (PVN). Fluorescence intensity was measured in young or old female rats that were subjected to the NMS protocol (red symbols; 3 h/day from postnatal days 3 to 12) or maintained under standard animal care over the same period (control; CTRL, blue symbols). Bar height indicates mean value ± SD. ANOVA results are reported under each figure.

In both parvocellular and magnocellular regions of the PVN, the intensity of synaptophysin and GFAP immunolabelling did not differ significantly between young and older females, regardless of NMS exposure (Figure 6a, b). Statistical analyses revealed no significant effects of age, NMS or their interaction on either marker. Thus, synaptic and astroglial protein expression in the PVN appears to remain stable across age and early life stress conditions.

### 3.6 The effects of NMS on ACE2 (but not ACE) activity are age‐specific

**FIGURE 7:**
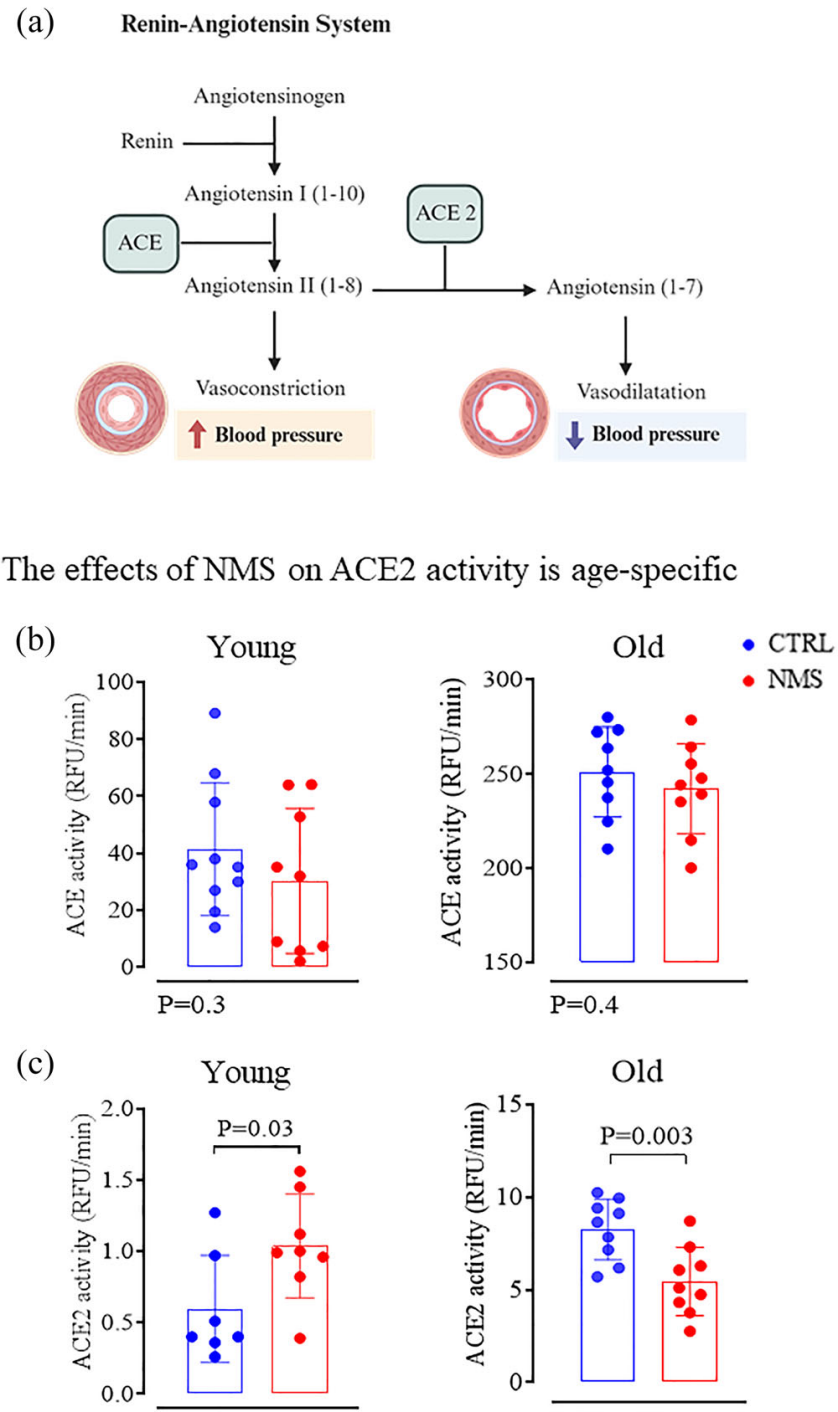
(a) Schematic representation of the main components of the renin–angiotensin system and its effect on blood vessels. (b, c) Comparison the angiotensin‐converting enzyme (ACE; left) (b) and ACE2 (c) activity in young (left) or old (right) female rats that were subjected to neonatal maternal separation protocol (NMS, red symbols; 3 h/day from postnatal days 3 to 12) or maintained under standard animal care over the same period (control; CTRL, blue symbols). Bar height indicates mean value ± SD. *P*‐values indicate results from unpaired *t*‐test.

**FIGURE 8:**
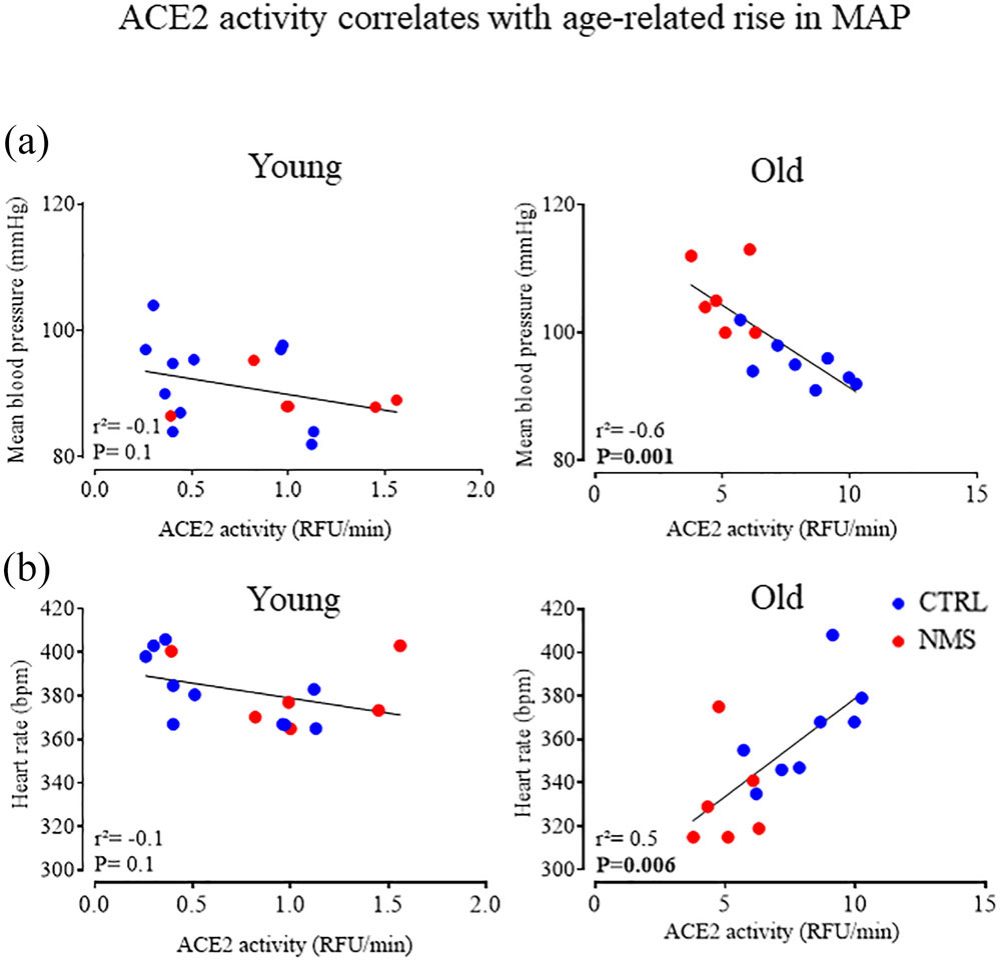
Correlation analyses comparing the effect of age on the relationships between ACE2 and (a) mean arterial pressure and (b) heart rate mean. Scatter plots are presented for young (left) and old (right) females. Data are reported for females subjected to neonatal maternal separation protocol (NMS, red symbols; 3 h/day from postnatal days 3 to 12) or maintained under standard animal care over the same period (control; CTRL, blue symbols). Coefficients of determination (*r* ^2^) and *P*‐values are reported separately for CTRL and NMS.

Figure 7a illustrates the main components of the renin–angiotensin system and highlights how ACE and ACE2 act on blood vessels. ACE activity did not differ between CTRL and NMS animals at either age (Figure 7b, left and right).  In contrast, ACE2 activity of young NMS rats was 42% higher than CTRLs (Figure 7c, left). This pattern reversed in old age, as the ACE2 activity values observed in NMS were now 35% lower than those of CTRLs (Figure 7c, right). The link between NMS‐related changes in ACE2 expression and MAP was evaluated with correlational analyses, and while no significant associations between ACE2 activity and MAP was observed in young females, a strong inverse correlation was observed in old animals (Figure 8a, left and right, respectively). The relationship between ACE2 and HR was also age‐specific but unlike MAP, the significant correlation observed in old females was positive (Figure 8b, left and right). ANCOVA revealed a significant main interaction between stress and ACE2 activity in predicting MAP (*F*_1,27_ = 9.9, *P* = 0.004).

## 4 DISCUSSION

Cardiovascular disease is a primary cause of death worldwide and high blood pressure results in approximately half of all cardiovascular‐related deaths (Di Cesare et al., 2024; Lindstrom et al., 2022). Because the risk of women developing hypertension augments significantly once they enter menopause (Drury et al., 2024; Wenger et al., 2018), elucidating the factors and mechanisms that predispose a subset of post‐menopausal women to develop this problem is essential to the development of more targeted preventive and therapeutic strategies. The results reported here make a significant contribution in that regard as they support the hypothesis that experiencing adversity during early life makes females more vulnerable to hypertensive trajectories following reproductive senescence. Furthermore, comparing the trajectories of CTRL versus NMS rats revealed two distinct mechanisms that likely contribute to this problem: a stress‐related increase in cardiovascular responsiveness to 17β‐oestradiol (E_2_) and ACE2.

### 4.1 Ovarian hormones and the age‐related increase in blood pressure

Young NMS females exhibited higher circulating E_2_ levels compared to CTRL, whereas the effects of NMS on progesterone levels were limited to a reduction in the metoestrus phase. These results align with observations by Amorim et al. (2011), who reported that adult female rats (120 days old) subjected to low maternal care had elevated circulating E_2_ with no changes in progesterone levels (Amorim et al., 2011). This is also consistent with reports indicating that early life stress enhances ovarian hormone secretion, likely through long‐lasting effects of stress on steroidogenesis and feedback regulation (Shors et al., 1999).

Considering that NMS augments MAP in young males (Genest et al., 2004), the lower MAP reported in NMS females of similar age was unexpected at first; however, this result is consistent with the higher E_2_ levels observed in NMS females. 17β‐Oestradiol (E_2_) reduces smooth muscle contractility, sympathetic activity and MAP in preeclamptic women (Babic et al., 2018), and we observed an inverse relationship between E_2_ and MAP in NMS females. The lack of correlation in CTRL points to a stress‐related increase in E_2_ sensitivity which is in line with the enhanced action of E_2_ supplementation on the ventilatory response to CO_2_ reported in NMS females (Tenorio‐Lopes et al., 2020). The underlying mechanism is unknown but could reflect an increase in the oestrogen receptor‐β (Erβ) and aromatase expression as reported in female rats following acute stress (Liu et al., 2011).

In mice, progesterone augments MAP by increasing mineralocorticoid receptor expression in endothelial cells (Faulkner et al., 2019). This mechanism likely contributes to the positive correlation between MAP and progesterone observed in NMS females but its role in the age‐related rise in MAP in stressed females is unlikely. By comparison with E_2_, the effect of NMS on progesterone levels was more variable and unlike MAP in NMS females, progesterone levels did not increase with age.

The rise in MAP observed in NMS females increased abruptly between adulthood and middle age and stabilized thereafter. Such pattern differs from the one reported in women as the incidence of hypertension rises progressively with age (Connelly et al., 2022). Accordingly, the reasons why MAP of old NMS females is not greater than those observed at middle age are not clear, but do not reflect a ‘ceiling effect’ as higher blood pressures have been reported in hypertensive rats (Reckelhoff et al., 2000).

### 4.2 Ageing trajectory of the hypothalamic–pituitary–adrenal axis

Exposure to stressful stimuli activates the PVN which then initiates and regulates neuroendocrine responses (Herman et al., 2003). As ACTH release is upstream in the signalling cascade, corticosterone levels generally follow ACTH. However, a dissociation between ACTH and corticosterone can occur (Aguilera, 1994) and the results observed in ageing females are in line with previous evidence from male studies showing that early‐life stress reduces adrenal sensitivity to ACTH stimulation, resulting in lower corticosterone output (Holubová et al., 2016).

In addition to its primary role in regulating the stress pathways, the PVN also exerts a ‘top down’ influence on the sympathetic system and cardiovascular function (Dampney et al., 2018; Faulkner et al., 2019). Within the PVN, neurons of the parvocellular region project to the autonomic structures of the brainstem and while the actions of E_2_ on the PVN are diverse and complex (Grassi et al., 2022), E_2_ supplementation attenuates cFos expression in the PVN (Gerrits et al., 2005). Thus, the elevated E_2_ observed in young NMS females may contribute to the lower density of FosB expressing cells in the parvocellular region of this age group, and the increased density in older animals may reflect the age‐related drop in E_2_ observed in this group. As changes in PVN activity can affect sympathetic tone, this may contribute to the age‐related changes in MAP observed in NMS females.

FosB isoforms show extraordinary long half‐lives (weeks), but their expression may no longer be detectable 1 year after NMS exposure (Nestler et al., 2001; Uchida et al., 2025). The stability of synaptophysin and GFAP levels in the PVN indicate that the number of synaptic vesicles or glia cells of PVN was unaffected by ageing such that the decrease in FosB expression most likely reflects a reduction in neuronal activity rather than a decline in network functionality. Thus, mechanisms other than sympathetic activity likely explain the age‐related rise in MAP of NMS females. While a reduction of E_2_’s relaxing action on blood vessels is a possibility that was not tested, the age‐related effects of NMS the renin–angiotensin system offer a plausible explanation.

### 4.3 Age‐related changes in the long‐term effects of NMS on ACE2 activity

Sex steroids affect various components of the renin–angiotensin system and thus contribute to many sex‐based differences in renal sodium handling and ultimately, blood pressure regulation (Drury et al., 2024). Within this system, ACE and ACE2 regulate angiotensin activity, and thus play important (and opposing) roles in the maintenance of vascular tone and blood pressure (Ji et al., 2020). Results from gonadectomy experiments show that sex hormones generally have a greater influence on ACE2 activity than ACE activity in rats (Dalpiaz et al., 2015; Melo Junior et al., 2020), which may explain why ACE (but not ACE2) was unaffected by NMS. E_2_ augments ACE2 expression in multiple tissues (Mompeón et al., 2016), and while our measurements were performed in plasma, the changes in ACE2 activity (but not ACE) parallel the effect of NMS on E_2_ levels. ACE2 promotes vasodilation and while the correlation between ACE2 and MAP was not significant in young females, the trend observed suggests that NMS‐related reduction in ACE2 activity contributes to the lower MAP measured in this group. ACE2 has been widely recognized as a protective factor in cardiovascular ageing and disease, and its reduction may exacerbate vascular resistance and sympathetic drive (Oudit et al., 2003; Úri et al., 2016). Furthermore, the positive correlation between ACE2 and HR in aged animals suggests broader roles for this enzyme in autonomic regulation, extending beyond vascular effects alone. These findings align with and extend previous evidence that, in males, neonatal stress sensitizes the renin–angiotensin system to angiotensin II, leading to exaggerated hypertension, tachycardia and vascular inflammation, despite normal baseline haemodynamics (Bertagnolli et al., 2016; Loria et al., 2010). The sum of these results reinforces the notion that the deleterious impact of early life stress on the renin–angiotensin system is an important mechanism in the long‐term cardiovascular vulnerability we observed in NMS females.

### 4.4 Limitations

While the longitudinal design across distinct reproductive stages is a strength of this study, it imposed several limitations. For instance, ACE and ACE2 and brain analyses were not performed in middle‐aged animals, such that our evaluation of females in the ‘perimenopausal’ transition was incomplete. The neuronal analyses were confined to the PVN, leaving the possibility of alterations in other brain regions open. Finally, the study lacks direct testing of mechanistic pathways; moreover, the absence of hormone replacement therapy testing in both groups limits our ability to draw more definitive conclusions. Despite these constraints, our findings strongly support the concept that early life stress imprints a latent cardiovascular vulnerability that is unmasked with reproductive senescence.

### 4.5 Conclusion and future directions

Our findings, therefore, bring valuable support to the ‘two‐hit hypothesis’ proposed by Loria, Ho and Pollock (Loria et al., 2013b) in which early life stress is the first hit that augments cardiovascular vulnerability, and experiencing a second environmental stressor later in life induces dysfunction. Here, we demonstrate that age‐related E_2_ reduction acts as the second hit that unmasks a pre‐hypertensive phenotype; NMS‐related changes in ACE2 activity likely contribute to this problem. As the CO_2_ response of NMS exposed animals is more sensitive to changes in ovarian hormones (Tenorio‐Lopes et al., 2020), the diversity of early life experiences provides a plausible explanation for the heterogeneity of the effects of loss of ovarian function on cardiovascular function in animals and humans. It may also explain the variability in the efficacy of hormone replacement therapy at alleviating menopause‐related health issues, including hypertension (Drury et al., 2024). Given that early‐life adversity can modify responses to various stressors later in life, it would be of considerable interest to examine age‐related changes in MAP among NMS and control females when exposed to additional relevant stressors, such as obesity or social isolation. Such an investigation would strengthen the foundational principles established in the present study.

## AUTHOR CONTRIBUTIONS

Danuzia Ambrozio‐Marques, Tim D. Ostrowski, Aline M. Arlindo de Souza and Richard Kinkead conceptualized and designed this study. Danuzia Ambrozio‐Marques, Loralie Mei Guay, Alicia A. Koogler, Aline M. Arlindo de Souza performed the experiments and analysed the data. Danuzia Ambrozio‐Marques and Richard Kinkead and wrote the paper. All authors reviewed and edited the manuscript. All authors have read and approved the final version of this manuscript and agree to be accountable for all aspects of the work in ensuring that questions related to the accuracy or integrity of any part of the work are appropriately investigated and resolved. All persons designated as authors qualify for authorship, and all those who qualify for authorship are listed.

## CONFLICT OF INTEREST

None declared.

## Data Availability

All data supporting the results are reported in the manuscript. Raw results can be provided upon request.
